# *CHRNA4* rs1044396 is associated with smoking cessation in varenicline therapy

**DOI:** 10.3389/fgene.2015.00046

**Published:** 2015-02-27

**Authors:** Juliana Rocha Santos, Paulo R. X. Tomaz, Jaqueline S. Issa, Tânia O. Abe, José E. Krieger, Alexandre C. Pereira, Paulo C. J. L. Santos

**Affiliations:** ^1^Laboratory of Genetics and Molecular Cardiology, Heart Institute, University of Sao Paulo Medical SchoolSão Paulo, Brazil; ^2^Smoking Cessation Program Department, Heart Institute, University of Sao Paulo Medical SchoolSão Paulo, Brazil

**Keywords:** varenicline, pharmacogenetic, smoking cessation, polymorphism, *CHRNA4*, *CHRNB2*

## Abstract

**Background:** The large individual variability in response to drugs for smoking cessation suggests that specific treatments can be more effective in particular subgroups of smokers. In the context of personalized medicine, the main aim of the present study was to evaluate whether the *CHRNA4* and *CHRNB2* polymorphisms are associated with response to smoking cessation therapies in patients from a smoker assistance program.

**Methods:** This cohort study enrolled 483 smoking patients who received behavioral counseling and drug treatment (varenicline, bupropion, and/or nicotine replacement therapy). Smoking cessation success was considered for patients who completed 6 months of continuous abstinence. Fagerström test for nicotine dependence (FTND) and Issa situational smoking scores were analyzed for nicotine dependence. The *CHRNA4* (rs1044396 and rs2236196) and *CHRNB2* (rs2072660 and rs2072661) polymorphisms were genotyped by high resolution melting analysis.

**Results:** Patients with rs1044396 CC genotype had lower success rate in treatment with varenicline (29.5%) compared with carriers of CT or TT genotypes (50.9%; *p* = 0.007, *n* = 167). The CT or TT genotypes were associated with higher odds ratio for success (OR = 1.67, 95% CI = 1.10–2.53, *p* = 0.02), in a multivariate model. We did not observe significant differences in the FTND and Issa scores according to the studied polymorphisms.

**Conclusion:** The *CHRNA4* rs1044396 is associated with smoking cessation in individuals on varenicline therapy. We suggest that this polymorphism influences the varenicline response, but replications of this finding are needed.

## INTRODUCTION

Smoking is one of the most important risk factors associated with the development of cardiovascular and respiratory diseases, besides being considered a leading cause of cancer death (MacLean and Chockalingam, 1999; Pappas et al., 2007; Oliveira et al., 2008; Roger et al., 2012; Jairam et al., 2013). Data showed that current smokers have an increased cardiovascular risk compared to former smokers even in subjects with a long and intense smoking history (Jairam et al., 2013). A current epidemiological study, involving 187 countries, reported a moderate reduction in the prevalence of smoking at the global level. Nevertheless, the number of smokers significantly increased from 721 million in 1980 to 967 million in 2012 (Ng et al., 2014).

Many smokers are aware of these harmful effects. However, only 4–7% of smokers are able to quit smoking without medication or formal treatment (Fiore et al., 2008; Centers for Disease Control and Prevention (CDC), 2010). First line drugs for smoking cessation approved by the FDA (US Food and Drug Administration) include nicotine replacement therapy (NRT), bupropion (a norepinephrine-dopamine reuptake inhibitor), and varenicline (a partial α4 and β2 agonist subunits of the nicotinic acetylcholine receptors). Varenicline has the greatest therapeutic efficacy in the smoking cessation process (Fiore et al., 2000, 2008).

The large individual variability in response to drugs for smoking cessation suggests that the treatment can be more effective in specific subgroups of smokers (Lerman et al., 2004; Swan and Lessov-Schlaggar, 2009; Quaak et al., 2014). In this way, the understanding of the factors that are involved in nicotine dependence (ND) and in the smoking cessation process is required to optimize this treatment. Studies demonstrated that genetic factors account for approximately 50% of the variance in smoking cessation success (Xian et al., 2003; Quaak et al., 2014). Also, these factors are less well known than the genetic factors that influence nicotine dependence (Xian et al., 2003; Lessov et al., 2004; Broms et al., 2006; King et al., 2012).

Several genetic association studies showed that polymorphisms in the *CHRNA4* and *CHRNB2* genes were associated with smoking initiation, ND, as well as with smoking cessation (Feng et al., 2004; Li et al., 2005; Ehringer et al., 2007; Hutchison et al., 2007; Conti et al., 2008; Breitling et al., 2009; Wessel et al., 2010; Chu et al., 2011; Han et al., 2011; King et al., 2012; Swan et al., 2012; Wei et al., 2012; Kamens et al., 2013). However, there are some phenotypes with controversial findings in the literature. The *CHRNA4* and *CHRNB2* encode the α4 and β2 subunits of the nicotinic acetylcholine receptors (nAChRs) which are the most abundant subunits in the brain and specific targets of the varenicline action (Zoli et al., 1998; Hogg et al., 2003; Coe et al., 2005). The α4 and β2 subunits are important targets of the nicotine action because the α4β2 nAChR are necessary and sufficient for nicotine reward, tolerance, and sensitization (Tapper et al., 2004).

In this context of personalized medicine, the main aim of the present study was to evaluate whether the *CHRNA4* and *CHRNB2* polymorphisms are associated with response to smoking cessation therapies in patients from a smoking assistance program.

## MATERIALS AND METHODS

### PATIENT SAMPLE

This cohort study enrolled 483 smoking patients from the PAF (Programa de Assistência ao Fumante/Smoker Assistance Program), Heart Institute (InCor), University of Sao Paulo Medical School, Sao Paulo, Brazil, between January, 2007 and September, 2013. The study protocol was approved by the Institutional Ethics Committee (0022/11).

The drug treatment consisted of an initial medical visit plus an average of four follow-up medical visits for 12 weeks. The follow-up was made by phone in patients who did not continue to come on scheduled medical visits. Clinical data and carbon monoxide concentration were collected in all visits. Demographic, socio-economic, and clinical data were acquired. Patients received behavioral counseling and drug treatment from physicians specialized in smoking cessation. Arbitrarily, bupropion plus NRT was prescribed for patients who smoked less than one cigarette pack per day; while varenicline was prescribed for patients who smoked one or more cigarette pack(s) per day, or who failed in previous attempts with bupropion plus NRT. Our indication to start the co-administration of bupropion and varenicline was if the patient did not achieve complete abstinence after 2 or 3 weeks of starting varenicline use, or if the patient achieved complete abstinent, but presented moderate, or intense discomfort abstinence symptoms. Continuous abstinence rate (CAR) was investigated after 6 months as of starting pharmacotherapy. Smoking status (outcome) was divided into: success group (patients who completed 6 months of CAR confirmed by carbon monoxide concentration), relapse group (patients who did not complete 6 months of CAR), and resistant group (patients who never achieved CAR after starting drug treatment; Issa et al., 2013, 2014).

We analyzed the Fagerström test for nicotine dependence (FTND) and Issa situational smoking score (Issa score). The FTND, a revised version of the Fagerström tolerance questionnaire, comprises six questions (ranging from 0 to 10 points) and the patients are grouped into five categories: 1–2 points = very low dependence; 3–4 points = low dependence; 5 points = medium dependence; 6–7 points = high dependence; and 8–10 points = very high dependence. The FTND is used in many countries as a cheap, non-invasive and easy way to assess ND (Fagerstrom et al., 1990). The Issa score comprises four questions (ranging from 0 to 4 points) with one point for each affirmative response. This score is based on psychoactive effects of nicotine in the process of cognition, attention, concentration, mood, well-being, and pleasure (Issa, 2012).

### GENOTYPING

Genomic DNA from subjects was extracted from peripheral blood following a standard salting-out procedure. Supplementary Figure S1 shows genotyping detected by polymerase chain reaction (PCR) followed by high resolution melting (HRM) analysis with the Rotor Gene 6000®; instrument (Qiagen, Courtaboeuf, France). The QIAgility®; (Qiagen, Courtaboeuf, France), an automated instrument, was used according to instructions to optimize the sample preparation step (Santos et al., 2011a,b). Amplifications of the fragments for the *CHRNA4* rs1044396 (c.C1629T), *CHRNA4* rs2236196 (c.G534A), *CHRNB2* rs2072660 (c.T313C), and *CHRNB2* rs2072661 (c.G472A) polymorphism were performed using the following primers sense and antisense: 5^′^- AGCCCTCTCCGTGCAAAT -3^′^ and 5^′^- CTTTGGTGCTGCGGGTCT -3^′^ (84 pairs base), 5^′^- ACCCTCTCCTAGCGAAGCAG -3^′^ and 5^′^- CAGGGTCCTTGAGCCTCTC -3^′^ (73 pairs base), 5^′^- AGCAAGGCTGCTAAGTGGAA -3^′^ and 5^′^- GACAATCCTGTCCCCTTCCT -3^′^ (89 pairs base), and 5^′^- CTGGCCTGACACAATGGTAG -3^′^ and 5^′^- GCTGCTGTCCACTCAAGCA -3^′^ (90 pairs base), respectively. A 40-cycle PCR was carried out with the following conditions: denaturation of the template DNA for first cycle of 94^∘^C for 120 s, denaturation of 94^∘^C for 20 s, annealing (59.6^∘^C for the rs1044396, 56.7^∘^C for the other) for 20 s, and extension of 72^∘^C for 22 s. PCR was performed with addition of fluorescent DNA-intercalating SYTO9®; (1.5 μM; Invitrogen, Carlsbad, CA, USA). In the HRM phase, the Rotor Gene 6000®; measured the fluorescence in each 0.1^∘^C temperature increase in the range of 70–88^∘^C. Melting curves were generated by the decrease in fluorescence with the increase in the temperature; and in analysis, nucleotide changes result in three different curve patterns (Supplementary Figure S1). Samples of the three observed curves were analyzed using bidirectional sequencing as a validation procedure (ABI Terminator Sequencing Kit®; and ABI 3500XL Sequencer®; – Applied Biosystems, Foster City, CA, USA; Santos et al., 2011a,b). The two methods gave identical results in all tests. The wild-type, heterozygous and homozygous genotypes could be easily discernible by HRM analysis. In addition, 6% of the samples were randomly selected and reanalyzed as quality controls and gave identical results.

### STATISTICAL ANALYSIS

Continuous variable data are presented as mean and SD and categorical variables as frequencies. Chi-square test was performed for the comparative analysis of categorical variables (general characteristics, smoking status rates, and categorized nicotine dependence scores) according to *CHRNA4* and *CHRNB2* polymorphisms. The Student’s *t*-test was used for comparing general characteristics and, FTND according to *CHRNA4* and *CHRNB2* polymorphisms. Logistic regression univariate and multivariate models were performed to evaluate the odds ratio (OR) for smoking cessation success. In the logistic regression, we showed results of the dominant and recessive models for the rs1044396 and for the rs2236196, respectively, based on findings from previous studies (Li et al., 2005; Markett et al., 2011; Tsai et al., 2012). Also, addictive models for the rs2072660 and rs2072660 were shown based on findings from previous studies (Ehringer et al., 2007; Wessel et al., 2010; Swan et al., 2012). Multiple linear regression models for FTND score were performed to evaluate the influence of the polymorphisms plus covariates. Linkage disequilibrium, Hardy–Weinberg equilibrium, and haplotype analysis were conducted with Haploview 4.0. All statistical analyses were carried out using SPSS software (v.16.0), with the level of significance set at *p* < 0.05.

## RESULTS

### GENERAL CHARACTERISTICS AND *CHRNA4* AND *CHRNB2* POLYMORPHISMS

**Table 1** shows general and clinical characteristics according to *CHRNA4* rs1044396 polymorphism in the overall patient sample and in patients treated with varenicline. We show the general characteristics among rs1044396 genotypes because it was the only one significantly associated with smoking cessation success. We did not find significant differences in the demographic and clinical data according to overall or varenicline groups.

**Table 1:** Demographic and clinical characteristics according to *CHRNA4* rs1044396 polymorphism.

	Overall (*n* = 483)		
	CC (*n* = 162)	CT or TT (*n* = 321)	*p*-value
Age (years)	52 ± 10	54 ± 23	0.27
Gender, female (%)	58.9	51.4	0.09
Self-declared race, White (%)	59.9	64.0	0.10
Scholarity, college (%)	27.2	27.4	0.55
Body mass index (Kg/m^2^)	27 ± 5	26 ± 5	0.06
FTND	6.9 ± 2.4	6.7 ± 2.5	0.66
FTND, ≥6 (%)	67.9	71.1	0.39
Issa score, 3 or 4 (%)	79.3	70.4	0.38
Hypertension (%)	42.6	40.2	0.61
Coronary artery disease (%)	13.0	17.4	0.20
Acute myocardial infarction (%)	16.0	19.0	0.43
Dyslipidemia (%)	43.8	36.4	0.12
Diabetes mellitus type 2 (%)	17.9	17.1	0.53
Obesity (%)	5.6	5.6	0.98
Depression (%)	19.1	20.6	0.71
Anxiety (%)	20.4	18.7	0.66
Chronic obstructive pulmonary disease (%)	11.7	16.8	0.14
Asthma (%)	2.5	1.9	0.66
Number of diagnosed diseases	2.3 ± 1.6	2.5 ± 1.7	0.43

	**Patients treated with varenicline (*n* = 167)**		
	**CC (*n* = 61)**	**CT or TT (*n* = 106)**	***p*-value**

Age (years)	52 ± 9	52 ± 11	0.84
Gender, female (%)	57.3	55.7	0.41
Self-declared race, White (%)	72.7	89.4	0.01
Scholarity, college (%)	32.8	34.9	0.78
Body mass index (Kg/m^2^)	27 ± 4	27 ± 5	0.46
FTND	7.2 ± 2.7	6.8 ± 2.6	0.45
FTND ≥6 (%)	73.8	74.7	0.83
Hypertension (%)	31.1	26.4	0.51
Coronary artery disease (%)	8.2	10.4	0.65
Acute myocardial infarction (%)	9.8	12.3	0.63
Dyslipidemia (%)	23.0	26.4	0.62
Diabetes mellitus type 2 (%)	13.0	17.9	0.10
Obesity (%)	0.0	3.8	0.13
Depression (%)	18.0	17.9	0.99
Anxiety (%)	13.1	11.3	0.73
Chronic obstructive pulmonary disease (%)	9.8	9.4	0.93
Asthma (%)	1.6	1.9	0.91
Number of diagnosed diseases	1.9 ± 1.5	1.8 ± 1.5	0.80

*FTND, Fagerström test for nicotine dependence (range from 0 to 10 points). Issa score, Issa situational smoking score (range from 0 to 4 points) was made for patients with FTND score ≤4 (*n* = 84). Issa score was not included for the group of patients with varenicline.*

The frequency of the *CHRNA4* rs1044396 T, *CHRNA4* rs2236196 G, *CHRNB2* rs2072660 T, and *CHRNB2* rs2072661 A alleles were 44.1, 36.8, 28.7, and 26.7%, respectively. The genotypic distributions for the rs1044396, rs2236196, rs2072660, and rs2072661 polymorphisms were in accordance with Hardy–Weinberg equilibrium (*X*^2^= 3.18, *p* = 0.05, *X*^2^= 1.12, *p* = 0.29, *X*^2^= 0.15, *p* = 0.70, and *X*^2^= 0.32, *p* = 0.57, respectively). Linkage disequilibrium analysis shows that the *CHRNA4* rs1044396 and rs2236196, and the *CHRNB2* rs2072660 and rs2072661 are in strong disequilibrium in our population (LD = 96, and LD = 89, *n* = 483; Supplementary Figure S2).

### SMOKING CESSATION SUCCESS ACCORDING TO *CHRNA4* rs1044396 POLYMORPHISM

**FIGURE 1:**
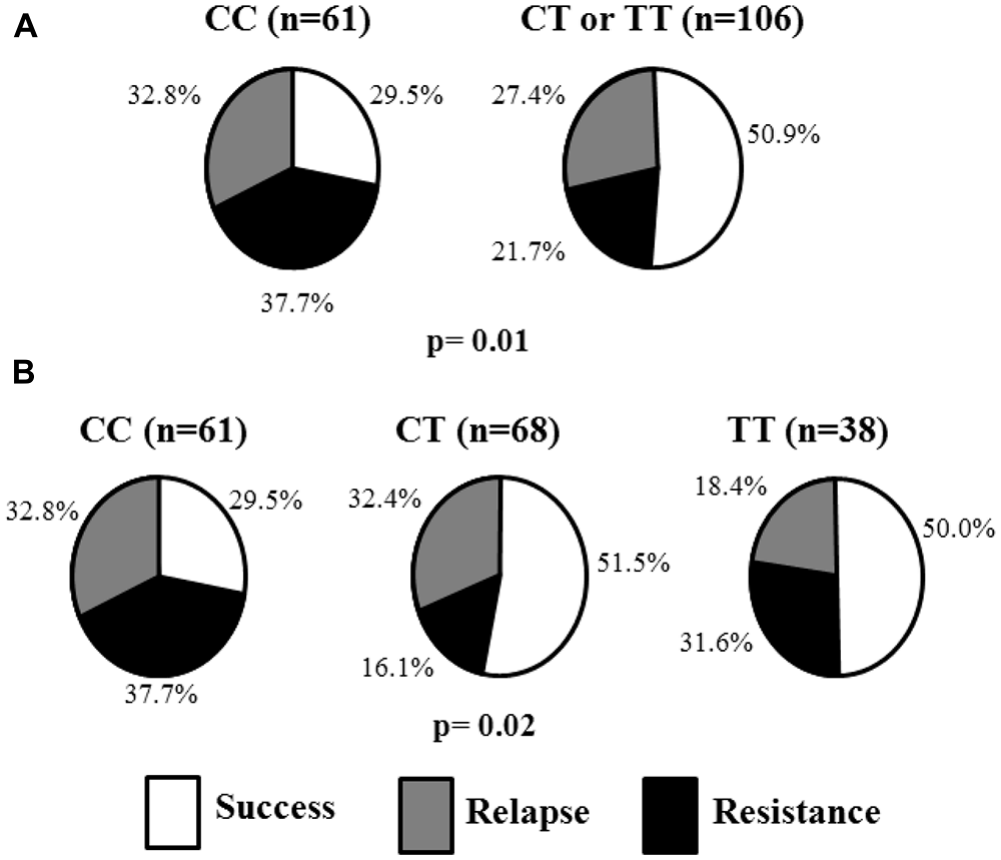
**Status of the patients treated with varenicline according to *CHRNA4* rs1044396 polymorphism. (A)** status according to dominant model. **(B)** status according to additive model.

**Table 2** shows smoking cessation success rates of patients according to prescribed drugs and *CHRNA4* rs1044396 polymorphism. Patients with the CC genotype had lower success rate when in treatment with varenicline (29.5%) compared with patients with CT or TT genotypes (50.9%; *p* = 0.007, *n* = 167). Also, **Figure 1** shows the distribution of the success, relapse, and resistance rates according to the genotypes in patients treated with varenicline. A significant difference in the smoking cessation rate was observed when separated into genotypic groups (CC = 29.5%, CT = 51.5%, TT = 50.0%; *p* = 0.02). No association was observed in other groups of drugs (varenicline plus bupropion, or bupropion plus NRT).

**Table 2:** Smoking cessation success rate of patients according to prescribed drugs and *CHRNA4* rs1044396 polymorphism.

	Success rate (%)		
Patient group	CC	CT or TT	*p*-value
Overall (*n* = 483)	37.0	46.4	0.05
Varenicline (*n* = 167)	29.5	50.9	0.007
Varenicline plus bupropion (*n* = 79)	40.0	50.0	0.41
Bupropion plus NRT (*n* = 237)	42.1	42.2	0.98

*NRT, nicotine replacement therapy (patch and/or gum).*

**Table 3** shows a logistic regression multivariate analysis for smoking cessation success in the patient group treated with varenicline. Patients with rs1044396 CT or TT genotypes were associated with higher OR for success (OR = 1.67, 95% CI = 1.10–2.53, *p* = 0.02), in a model including sex, age, race, and FTND score. In a logistic regression univariate analysis, CT or TT genotypes were associated with OR for success (OR = 2.48, 95% CI = 1.27–4.84, *p* = 0.008).

**Table 3:** Logistic regression multivariate analysis for smoking cessation success in the patients submitted to varenicline therapy (*n* = 167)

Variables	OR	95% CI	*p*-value
CT or TT genotypes for the *CHRNA4* rs1044396	1.67	1.10–2.53	0.02
Gender (male)	1.17	0.79–1.73	0.44
Age	1.01	0.97–1.05	0.31
Self-declared race (White)	1.07	0.70–1.61	0.77
FTND Score	0.96	0.89–1.05	0.38

*FTND, Fagerström test for nicotine dependence.*

In an analysis within the group of the White patients (*n* = 133; HWE *X*^2^ = 2.68; HWE *p*-value = 0.10), we also observed the same significant findings. Patients with the CC genotype had 30.0% of success with varenicline treatment, while patients with CT or TT genotypes had 51.6% (*p* = 0.02). In the logistic regression multivariate analysis, rs1044396 CT or TT genotypes were associated with higher OR for success (OR = 1.97, 95% CI = 1.16–3.34, *p* = 0.01).

### SMOKING CESSATION SUCCESS ACCORDING TO *CHRNA4* rs2236196, *CHRNB2* rs2072660, AND *CHRNB2* rs2072661 POLYMORPHISMS

Smoking cessation success rate did not show significant differences among rs2236196, rs2072660, and rs2072661 genotypes for all drug groups, even in a multivariate model.

For patient group with varenicline, the OR for success was of 1.15 (95% CI = 0.61–2.17, *p* = 0.66) for the variable *CHRNA4* rs2236196. Patients with AA, AG, or GG genotypes had 44.2, 46.3, and 30.4% of success during the varenicline therapy (*p* = 0.40).

For the *CHRNB2* rs2072660 and rs2072661polymorphisms, we found ORs of 1.01 (95% CI = 0.97–1.05, *p* = 0.66) and 1.08 (95% CI = 0.59–1.98, *p* = 0.81) for success in patients with varenicline, respectively. Patients with rs2072660 TT, TC, or CC genotypes had 41.9%, 44.3%, and 46.2% of success during the varenicline therapy (*p* = 0.94). Patients with rs2072661 GG, GA, or AA genotypes had 40.9, 46.8, and 41.7% of success during the varenicline therapy (*p* = 0.76).

### HAPLOTYPE ANALYSIS

The T-A haplotype for the *CHRNA4* was associated with smoking cessation success (OR = 1.33, 95% CI = 1.03–1.72, *p* = 0.03) in a multivariate analysis, while no association was found for the C-G and C-A haplotypes. For the *CHRNB2* C–G, T–A, T–G, and C–A haplotypes, no association was found with smoking cessation success (*p*-values: 0.96, 0.56, 0.32, and 0.57, respectively).

### FTND AND ISSA SCORES ACCORDING TO *CHRNA4* AND *CHRNB2* POLYMORPHISMS

We did not observe significant differences in the FTND and Issa scores according to rs1044396 polymorphism in the overall patient group (**Table 1**). In addition, studied polymorphisms were not associated with FTND score in multiple linear regression models (Supplementary Table S1).

## DISCUSSION

Several studies have indicated the *CHRNA4* and *CHRNB2* genes as strong candidates for the understanding of genetic factors related to ND and smoking cessation success (Li et al., 2005; Ehringer et al., 2007; Conti et al., 2008; Breitling et al., 2009; Wessel et al., 2010; Chu et al., 2011; King et al., 2012; Swan et al., 2012; Wei et al., 2012; Kamens et al., 2013). In our study, the main finding is the association of *CHRNA4* rs1044396 polymorphism with the smoking cessation success in patients treated with varenicline. No genetic association was identified for patients treated with bupropion and NRT. Corroborating our findings, King et al. (2012) also identified significant association of *CHRNA4* variants with varenicline response, but not with bupropion. These findings might likely occur because the *CHRNA4* gene encodes receptors which are the specific targets of varenicline action (King et al., 2012). Probably, in this present study, we did not find statistical difference in the success rate according to the rs1044396 among patients treated with varenicline plus bupropion because of low statistical power due to small sample size. Also, patients with CC genotype treated with varenicline plus bupropion did not have low success rate as in the case of patients with CC genotype treated with varenicline.

We used a dominant model for the *CHRNA4* rs1044396 according to previous studies (Markett et al., 2011; Tsai et al., 2012). We observed that patients treated with varenicline carrying TT or CT genotypes had an OR of 2.18 for smoking cessation success compared with patients carrying CC genotype. Even in an analysis using only self-reported White patients, our result was significant. Corroborating our finding, previous studies showed the association of the *CHRNA4* rs1044396 T allele with a protective effect in different phenotypes, such as: lower levels of depression, anxiety, and emotional instability, and lower propensity for ND (Feng et al., 2004; Breitling et al., 2009; Markett et al., 2011; Tsai et al., 2012).

To date, there are very few studies on the pharmacogenetic of varenicline (King et al., 2012; Swan et al., 2012). King et al. (2012) provided evidence that multiple genetic *loci* contribute to smoking cessation and therapeutic response. They also suggested that different *loci* are associated with varenicline *vs.* bupropion responses. Swan et al. (2012) showed the association of the *CHRNB2* variants with nausea, an adverse effect which is reported to be the most common reason for discontinuation of varenicline. They concluded that further pharmacogenetic investigations are warranted. Regarding the functional implication of the *CHRNA4* rs1044396 polymorphism, it is still not well established. Winterer et al. (2011) made a functional study for the *CHRNA4* rs1044396 polymorphism introducing a complementary DNA with a haplotype containing the rs1044396 into Xenopus oocytus expressing human α4β2 nAChR. They measured the electrophysiological response of the receptors under increasing concentration of acetylcholine and observed that the number of α4β2 nAChRs in the high-affinity state was different among haplotypes (Winterer et al., 2011). The high-affinity state indicates generally that a relatively low concentration of a ligand is sufficient to fully occupy a ligand-binding site and trigger a physiological response (Winterer et al., 2011; Greenwood et al., 2012). Thus, we suggest that this functional finding might be, at least partially, responsible for the association of rs1044396 with smoking cessation success in our scenario. In addition, the rs1044396 alleles can be in linkage disequilibrium with haplotype blocks as reported by Feng et al. (2004; rs2273504, rs2273502, rs1044396, rs1044397, rs3827020, and rs2236196) and by Han et al. (2011; rs2236196, rs3787138, rs10443196, rs1044394, and rs6010918).

Markou et al. (1998) and Paperwalla et al.’s (2004) studies showed that depression and anxiety are both highly comorbid with nicotine dependence. Thus, further studies could evaluate all these different endophenotypes in an attempt to elucidate the mechanisms by which *CHRNA4* polymorphisms might modulate psychiatric disorders, ND, and smoking cessation (Breitling et al., 2009).

Regarding the *CHRNA4* rs2236196, *CHRNB2* rs2072660, and *CHRNB2* rs2072661 polymorphisms, we did not identify any significant association with the response to smoking cessation therapies. A study involving 925 smokers treated with NRT from the United Kingdom did not find any association of the rs2236196 and rs2072660 with smoking cessation, but they also did not find an association of the rs1044396 with NRT response (Spruell et al., 2012). In a clinical trial of varenicline, King et al. (2012) found a significant association between rs2236196 G allele with continuous abstinence from 9 to 12 weeks. Hutchison et al. (2007) studying smokers of European ancestry, reported that the rs2236196 G allele was associated with differential treatment response in a trial comparing different forms of nicotine therapy. Another study showed associations of the rs2072660 with the abstinence rate in patients treated with bupropion (Conti et al., 2008) and of the rs2072661 with quitting success in response to nicotine patch (Perkins et al., 2009).

Regarding FTND and Issa scores, no significant association was found for the *CHRNA4* or *CHRNB2* studied polymorphisms. Similarly, other studies did not find an association between these polymorphisms with ND (Feng et al., 2004; Li et al., 2005; Etter et al., 2009; Han et al., 2011). However, some studies reported a significant association of the *CHRNA4* rs1044396 and rs2236196 polymorphisms with ND. Breitling et al. (2009) showed that the C allele for rs1044396 and G allele for rs2236196 may be associated with higher risk for ND in Germans. Li et al. (2005) showed association of the rs2236196 with ND in African–American carriers of the A allele. An interesting observation is that the minor allele for the rs2236196 differs among ethnic groups. The G is the minor allele in Europeans and the A is the minor allele in African–Americans. This difference in allelic frequency may help to explain the somehow controversial findings among ethnic backgrounds (Li et al., 2005). For the *CHRNB2*, Wessel et al. (2010) observed association of the rs2072660 and rs2072661 with mean of FTND score. Another interesting study provided evidence on the presence of gene–gene interaction among the four genes (*CHRNA4*, *CHRNB2*, *BDNF*, and *NTRK2*) in affecting ND (Li et al., 2008).

There are some limitations in our study. First, our sample size of patients treated with varenicline is relatively small and, consequently, our statistical power is reduced and a spurious association effect is possible. However, we were able to identify significant differences among genotypes, even including potential confounders. Second, we were not able to evaluate the main finding in the non-White group because of the number of patients. Our main finding remains significant even in an analysis of the self-reported White patients. The self-reported race/color classification is the most applied in the clinical routine and some studies showed that it is a good proxy of genetic ancestry information (Pena et al., 2011; Soares et al., 2012). Third, the range in the FTND score is small in this patient cohort because most of the patients who attended PAF were classified as having moderate or strong dependency. Fourth, we used multivariate models with the available variables, but we were not able to add other biological aspects or environmental factors which could be important, such as functionality of the receptors, depression and motivation.

In conclusion, the *CHRNA4* rs1044396 is associated with smoking cessation in varenicline therapy. We suggest that this polymorphism influences the varenicline response, but replications of this finding are needed.

## Conflict of Interest Statement

The authors declare that the research was conducted in the absence of any commercial or financial relationships that could be construed as a potential conflict of interest.
