# Supplementary material for: CHRNA4 rs1044396 is associated with smoking cessation in varenicline therapy
Source: Front Genet. 2015 Feb 27;6:46. doi: 10.3389/fgene.2015.00046 (PMC4343187; doi:10.3389/fgene.2015.00046)
Supplement: Supplementary file 1 [file data_sheet_1.docx]

**SUPPLEMENTARY MATERIAL**

**Table**

**Supplementary table 1.** Variables of a multiple linear regression model for FTND score

| **Variable** | **β coefficient (standard error)** | **p value** |
| --- | --- | --- |
| **FTND** | | |
| **Number of variant allele for the *CHRNA4* rs1044396** | -0.04 (0.2) | 0.82 |
| **Age** | 0.001 (0.006) | 0.86 |
| **Sex (male)** | 0.3 (0.2) | 0.88 |
| **Race (White)** | 0.1 (0.3) | 0.74 |
| **Scholarity** | -0.1 (0.2) | 0.42 |
| **FTND** | | |
| **Number of variant allele for the *CHRNA4* rs2236196** | -0.2 (0.2) | 0.29 |
| **Age** | 0.001 (0.006) | 0.93 |
| **Sex (male)** | 0.3 (0.2) | 0.90 |
| **Race (White)** | 0.05 (0.3) | 0.86 |
| **Scholarity** | -0.1 (0.2) | 0.41 |
| **FTND** | | |
| **Number of variant allele for the *CHRNB2* rs2072660** | 0.03 (0.2) | 0.89 |
| **Age** | 0.001 (0.006) | 0.88 |
| **Sex (male)** | 0.6 (0.3) | 0.33 |
| **Race (White)** | 0.09 (0.3) | 0.76 |
| **Scholarity** | -0.1 (0.1) | 0.42 |
| **FTND** | | |
| **Number of variant allele for the *CHRNB2* rs2072661** | 0.1 (0.2) | 0.49 |
| **Age** | 0.001 (0.006) | 0.84 |
| **Sex (male)** | 0.6 (0.3) | 0.22 |
| **Race (White)** | 0.07 (0.3) | 0.82 |
| **Scholarity** | -0.1 (0.2) | 0.45 |

Numbers of variant allele for *CHRNA* rs10436196 were 0, 1 or 2 for CC, CT, or TT, respectively.

For *CHRNA4* rs2236196 were 0, 1 or 2 for AA, AG or GG, respectively.

For *CHRNB2* rs2072660 were 0, 1 or 2 for TT, TC or CC, respectively.

For *CHRNB2* rs2072661 were 0, 1 or 2 for GG, GA or AA, respectively.

FTND: Fagerström test for nicotine dependence (n=483).

**Figure**
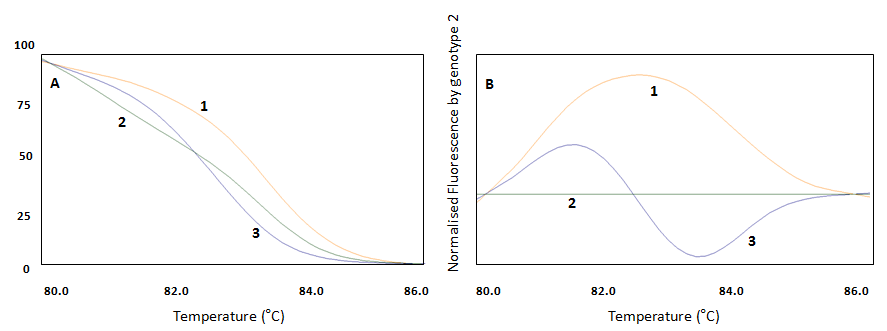


**Supplementary Figure 1. Graphs of the *CHRNA4* rs1044396 genotyping.** Nucleotide changes results in different curve patterns using high resolution melting analysis. A: Graph of normalized fluorescence by temperature. B: Graph of normalized fluorescence (based on genotype 2) by temperature. 1: wild-type (CC); 2: heterozygous (CT); 3: variant homozygous (TT).


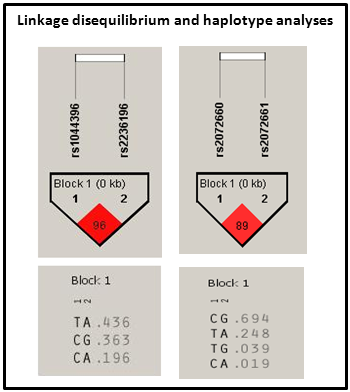


**Supplementary Figure 2**. Linkage disequilibrium and haplotype analysis for the *CHRNA4* and *CHRNB2* polymorphisms in the patients submitted to treatment for smoking cessation (n=483).
